# Nutrition of Infants and Young Children in Poland – Pitnuts 2016

**DOI:** 10.34763/devperiodmed.20172101.1328

**Published:** 2017-05-29

**Authors:** Halina Weker, Marta Barańska, Agnieszka Riahi, Małgorzata Strucińska, Małgorzata Więch, Grażyna Rowicka, Hanna Dyląg, Witold Klemarczyk, Agnieszka Bzikowska, Piotr Socha

**Affiliations:** 1 Nutrition Department, Institute of Mother and Child, Warsaw, Poland; 2 Human Nutrition Department, Faculty of Health Sciences, Medical University of Warsaw, Warsaw Poland; 3 Early Psychological Intervention Department, Institute of Mother and Child, Warsaw, Poland; 4 Gastroenterology, Hepatology, Nutrition Disorders and Paediatric Department, Children’s Memorial Health Institute, Warsaw, Poland

**Keywords:** infants, children 1-3 years old, breastfeeding, complementary feeding, diet, niemowlęta, dzieci 1-3 lata, karmienie piersią, żywność uzupełniająca, sposób żywienia

## Abstract

The study evaluating the feeding practices and the nutritional status of children aged 5 to 36 months in a general, Polish, representative population (n=1059) was carried out from May to July 2016. The aim of this study was to evaluate the feeding practices in children aged 5 to 36 months with regard to models of safe nutrition on the basis of the outcome of the population study performed in 2016. The data obtained show that the feeding practices in children in their first year of life do not meet the guidelines presented in the model of safe nutrition, particularly in matters of timing of complementary feeding introduction and food choice. The analysis of nutrient profile in toddlers’ diets indicated the differentiated energy and protein intake is significantly higher than population norms (EAR/AI). It is necessary to modify the nutrition of infants and young children through a better selection of products. Nutritional practice should always be monitored and modified according to the model of safe nutrition as part of medical nutritional counselling. More educational efforts are required to increase the awareness of the relation between the diet and nutritional status of young children among healthcare professionals.

## Streszczenie

W 2016 roku w okresie od maja do lipca, przeprowadzono na ogólnopolskiej, reprezentatywnej próbie dzieci (n=1059) w wieku od 5 do 36 miesiąca, badania dotyczące oceny ich sposobu żywienia i stanu odżywienia. Celem pracy była ocena postępowania żywieniowego u dzieci w wieku od 5 do 36 miesiąca życia w Polsce na podstawie wyników badania populacyjnego przeprowadzonego przez zespół z Instytutu Matki i Dziecka w 2016 roku w odniesieniu do modeli bezpiecznego żywienia. Z przeprowadzonej analizy wynika, że sposób żywienia dzieci w 1. roku życia, w tym czas rozszerzania diety, dobór produktów różnił się istotnie od schematu/zaleceń modelu bezpiecznego żywienia. Natomiast analiza profilu składników odżywczych w dietach dzieci w wieku poniemowlęcym wykazała zróżnicowaną wartość energetyczną oraz znacząco wyższą podaż białka w odniesieniu do norm populacyjnych (EAR/ AI). Modyfikacja żywienia niemowląt i małych dzieci poprzez korzystniejszy dobór produktów jest konieczna. Monitorowanie i korygowanie sposobu żywienia małych dzieci, w odniesieniu do zaleceń modelu bezpiecznego żywienia, powinno być stałym elementem porad medyczno-żywieniowych. Z uwagi na zależność stanu odżywienia dziecka od jego sposobu żywienia istnieje potrzeba edukacji żywieniowej personelu medycznego.

## Introduction

The adequate nutrition of young children plays a crucial role in their optimal mental and physical development. This happens due to the reduction of risk for several diseases, including respiratory and digestive tract infections, as well as diet-related diseases, such as obesity, type 2 diabetes mellitus and cardiovascular disease [1, 2, 3]. Nutrition affects the maturation and normal functioning of all systems of the child’s body during the entire developmental period. It applies especially to the so-called critical periods, including the prenatal period and the first two years of life [1].

Epidemiological observations and clinical trials concerning the impact of nutrition on the nutritional status indicated that incorrect nutrition, including inadequate profile of nutrients in the child’s diet, leads to disorders of the nutritional status and increases the risk of nutritional deficiencies [1, 4, 5, 6, 7, 8, 9]. Four basic elements describing the rules of adequate nutrition have been identified in the models of safe nutrition, which should be understood as a system of nutritional recommendations developed on the basis of objectivised studies in the field of medical nutrition. These include: the organisation of the meals/eating frequency, selection of products in daily diet, energy and nutrient intake meeting the child’s requirement, and other factors, including cultural patterns, familial, environmental and behavioural determinants, dietary habits, as well as physical activity [10].

The Polish standard of feeding children in their first year of life (tab. I), which was developed in 2014 and modified in 2016 by a group of experts appointed by the national consultant in paediatrics, promotes breastfeeding and presents guidelines for feeding breastfed and non-breastfed infants. It also reveals the suggested number and types of meals and the portion size the child should receive at a given age [5, 11, 12, 13]. The gradual accustoming the child to new products and encouraging it to try new $avours and textures of food has a positive impact on the process of expanding the diet. The standard contains information on what kind of food the child should receive and when, yet it is the child, who decides how much he/she eats.

**Table I:** Nutrition of children in the first year of life. Update 2016 Tabela I. Schemat żywienia dzieci w 1. roku życia. Aktualizacja 2016.

Age (months) *Wiek (miesiące)*	Abilities *Umiejętności*	Number of meals per day (approximate) *Liczba posiłków w ciągu dnia (orientacyjna)*	Ration size (ml) (approximate) *Wielkość porcji (ml) (orientacyjna)*		Meals type and consistency *Rodzaj i konsystencja pokarmów*	Examples^2^ *Przykłady pokarmów^2^*
1	Suction and swallowing Ssanie *i połykanie*	7^*^	110		Liquids / *Płyny*	Mother's milk or infant formula *Mleko matki lub mleko modyfikowane*
2-4		6*	120-140			
5-6	Initial mincing of meals with the tongue *Początkowe rozdrabnianie pokarmów językiem* Strong suction reaction *Silny odruch ssania* Pushing the food out of the mouth with tongue (temporary reaction) *Wypychanie jedzenia z ust za pomocą języka (reakcja przejściowa)* Opens mouth when the spoon is brought closer *Otwieranie ust przy zbliżaniu łyżeczki*	5*	150-160	Breastfeeding or formula feeding^1^ *Karmienie piersią lub mlekiem modyfikowanym1*	Smooth purée *Gładkie purée* 4 dairy ** meals 4 *posiłki mleczne*	Cooked and mixed vegetables (eg. carrot) or fruits (e.g. apple, banana), meat, eggs, or potato purée, gluten-free infant cereals *Gotowane miksowane warzywa (np. marchew) lub owoce (np. jabłko, banan), mięso, jaja lub purée ziemniaczane, kaszki/kleiki bezglutenowe* Cereal products, including small amounts of gluten in any period after the child reaches the age of 4 months (17 weeks of life), until the age of 12 months *Produkty zbożowe, w tym gluten w małych ilościach w dowolnym okresie po ukończeniu 4 m.ż. (17. tyg.ż.) do 12 m.ż*.
7-8	Drawing food with the lips from the spoon *Pobieranie pokarmu z łyżeczki wargami* Biting, chewing, and lateral tongue movements *Gryzienie, żucie, ruchy języka na boki* Develops the abilities and coordination to eat independently *Rozwój umiejętności i koordynacji umożliwiających samodzielne jedzenie*	5*	170-180	Breastfeeding or formula feeding1 *Karmienie piersią lub mlekiem modyfikowanym1*	Increased diversity of minced or chopped products *Zwiększona różnorodność rozdrobnionych lub posiekanych pokarmów* Products given to child's hands *Produkty podawane do ręki* 3 dairy meals AT the age of 7-8 months *3 posiłki mleczne od 7-8. m.ż*.	Mixed / finely chopped meat and fish *Zmiksowane/drobno posiekane mięso, ryby* Mashed, cooked vegetables and fruits *Rozgniecione gotowane warzywa i owoce* Chopped fresh vegetables and fruits (e.g. apple, pear, tomato) *Posiekane surowe warzywa i owoce (np. jabłko, gruszka, pomidor)* Soft parts of vegetables, fruits, meat given to the hand *Miękkie kawałki/cząstki warzyw, owoców, mięsa podawane do ręki* Cereals, bread *Kasze, pieczywo* Full fat cow's milk^3^ after the 11-12 months of life *Pełne mleko krowie3 po 11-12. m.ż*. Natural yoghurt, cheese, kefir *Jogurt naturalny, sery, kefir*
9-12		4-5*	190-220			
**PARENT/GUARDIAN decides WHAT, WHEN and HOW the child eats, CHILD decides, WHETHER and HOW MUCH to eat. *RODZIC/OPIEKUN decyduje, CO dziecko zje, KIEDY i JAK jedzenie będzie podane. DZIECKO decyduje, CZY posiłek zje i ILE zje***.						

* Estimated number of formula-fed infants, it is possible that the number of meals is greater in breastfed children, resulting from breastfeeding episodes./ *Orientacyjna liczba posiłków u niemowląt karmionych sztucznie; u niemowląt karmionych naturalnie dopuszczalna jest większa liczba posiłków wynikająca z przystawiania dziecka do piersi. ^1^ Exclusive breastfeeding for the first 6 months of life. /^1^ *Wyłączne karmienie piersią przez pierwszych 6 m.ż*. ^2^ The milk is given from the breast, bottle with nipple or open mug. Other food is given using a spoon. / ^2^ *Mleko podawane jest z piersi, butelki ze smokiem lub otwartego kubka. Pozostałe pokarmy podajemy łyżeczką*. ^3^ Small amounts may be used to prepare complementary products, yet cow's milk should not be used as the main milk product for the first 12 months of life. /^3^ *Małe ilości można stosować do przygotowania pokarmów uzupełniających, ale mleko krowie nie powinno być stosowane jako główny produkt mleczny przed 12. m.ż*. DRINKS: water as needed. Juices (100, high-pulp, no sugar added, pasteurised), maximum 150 ml/day (the ration is calculated along with the amount of consumed fruits) Vitamin D and K supplementation according to the recommendations. */NAPOJE: do picia podajemy wodę bez ograniczeń. Soki (100, przecierowe, bez dodatku cukru, pasteryzowanej w ilości maksymalnie do 150 ml na dobę (porcja liczona razem z ilością spożywanych owoców). Suplementacja witaminy D i K zgodnie z rekomendacjami*.

The recommendations concerning post-infancy nutrition were developed based the objectivised scientific studies evaluating the impact the nutrition in the first two years of life has on the nutritional status of the child. The results indicate that it is most beneficial for the health and development of the child to continue breastfeeding until approx the. 12th month of life. Such feeding practice may also be used in the second and third year of life, as long as the mother and child wish. No upper age limit for breastfeeding has been established [13, 14].

## Aim

The aim of this study was to evaluate the feeding practices in children aged 5 to 36 months with regard to models of safe nutrition on the basis of the outcome of the population study performed in 2016.

## Material and Methods

The study evaluating the feeding practices and the nutritional status of children aged 5 to 36 months in a general, Polish, representative population (n=1059) was carried out from May to July 2016. The trial was performed as part of project No. 161/2016 entitled „Comprehensive evaluation of diet of children aged 5 to 36 months - nation-wide Polish trial 2016”, (pol. “Kompleksowa ocena sposobu żywienia dzieci w wieku od 5 do 36 miesiąca życia – badanie ogólnopolskie 2016 rok”) financed by the Nutricia Foundation.

The sample selection and parents/caregivers enrolment was performed by the research provider (TNS Polska). The children were randomly selected for the study using personal identification numbers (PESEL). The subjects came from all over Poland, so that appropriate territorial representativeness was obtained. Two subgroups were distinguished - children in their first year of life (n=447) and children in their second and third year of life (n=612).

The nutritional status was evaluated based on anthropometric features and indices - body weight [kg], body length/height [m] and body weight-for-height ratio standardized according to the reference WHO growth charts [15]. The anthropometric measurements were performed by the medical personnel or trained interviewers/dieticians with regard to the selected methodology [15, 16].

The feeding practices were evaluated by the questionnaire method (original questionnaire, including 3-day record of children’s diet carried out by the parents/caregivers according to the directions given). Based on the diet records we estimated the consumption of the products and the nutritional value of the diets was calculated using the “Dieta 5” nutritional computer programme [17, 18]. The results obtained were compared with age-adjusted nutritional recommendations – for infants aged 5-12 months and children aged 13-36 months [19].

The distribution of variables was analysed and the appropriate descriptive statistics were calculated (medians and interquartile ranges).

## Results

### Study group characteristics

There were 50.6% of boys and 49.4% of girls in the subgroup of children aged 5-12 months (n=447), whereas the number of boys and girls in the 13-36 months subgroup (n=612) was equal.

The Table II presents the study group characteristics. Most of the children lived in cities. Their parents had mostly higher education and it was the mothers who had higher education more frequently.

**Table II:** Characteristics of the groups of children aged 5 to 36 months (n=1059). Tabela II. Charakterystyka badanej grupy dzieci w wieku od 5 do 36 miesiąca życia (n=1059).

Variables *Zmienne*	Infants 5-12 months old (n=447)		Children 13-36 months old (n=612)	
**	*Niemowlęta 5-12 m.ż. (n=447)*		*Dzieci 13-36 m.ż. (n=612)*	
**Children’s age [months]**				
***Wiek dzieci*** *[miesiące]*				
** median (range ** 1-3 quartile) *mediana (zakres 1-3* *kwartyl)*	7.7 (5.8-10.2)		24.1 (17.8-30.0)	
**Sex [%]**				
***Płeć [%]***				
boys *chłopcy*	49.4		50.0	
** girls dziewczynki	50.6		50.0	
**Parents’ education [%] *Wykształcenie rodziców [%]***	**Mother *Matka***	**Father *Ojciec***	**Mother *Matka***	**Father *Ojciec***
Primary				
*podstawowe*	2.0	4.5	4.7	3.8
vocational				
*zawodowe*	13.4	22.6	12.6	22.2
secondary				
*średnie*	38.9	38.0	35.0	36.6
university				
*wyższe*	45.6	29.1	47.4	29.2
**Place of residence [%]**				
***Miejsce zamieszkania [%]***				
Agglomerations *aglomeracje*	13.0		12.3	
** large city *miasto duże*	13.4		13.1	
city *miasto*	10.1		8.8	
** town *miasto małe*	25.1		25.3	
countryside *wieś*	38.5		40.5	
**BMI [% of parents]**	**Mother**	**Father**	**Mother**	**Father**
***BMI [%rodziców]***	***Matka***	***Ojciec***	***Matka***	***Ojciec***
underweight (BMI<18,5)				
*niedowaga (BMI<18,5)*	5.1	0.7	4.6	0.2
normal (BMI≥18,5 and <25)				
*norma (BMI≥18,5 i <25)*	62.9	32.2	61.9	29.6
overweight (BMI≥25 and <30)				
*nadwaga (BMI≥25 i <30)*	21.7	45.9	22.2	47.9
obesity (BMI≥30)				
*otyłość (BMI≥30)*	9.8	14.1	9.0	12.9

### Infancy

The nutritional status of the children was evaluated based on the standardized weight-for-height ratio with regard to cut-off points established by the WHO [15]. The body weight of 67.6% of the infants was normal (the value of the analysed ratio ranged from -2SD to +1SD); 17.9% of the infants were overweight or at risk of becoming overweight, whereas 14.5% showed underweight (tab. III).

**Table III:** Nutritional status of the infants defined by the weight-for-height ratio – nation-wide, Polish, representative sample. Tabela III. Stan odżywienia badanych niemowląt określony poprzez znormalizowany wskaźnik masa ciała do długości/wysokości ciała – próba ogólnopolska, reprezentatywna.

Nutritional status *Stan odżywienia*	Cut-off points Weight-for-height z-score (acc. to WHO) *Punkty odcięcia Weight-for-height z-score (wg WHO)*	Infants 5-12 months old (n=447) *Niemowlęta 5-12 miesięcy (n=447)*	
		N	%
Possible risk of overweight *Możliwe ryzyko nadmiaru masy ciała*	>1 SD to 2 SD	61	13.7
Overweight/Nadwaga	>2 SD to 3 SD	13	2.9
Obese/Otyłość	>3 SD	6	1.3
Total/*Łącznie*		80	17.9
Wasted *Niedobór masy ciała do długości/wysokości ciała*	<-2 SD to -3 SD	42	9.4
Severely wasted *Znaczny niedobór masy ciała do długości/wysokości ciała*	<-3 SD	23	5.1
Total/*Łącznie*		65	14.5
** Normal nutritional status *Prawidłowy stan odżywienia* **	≥-2 SD to +1 SD	302	67.6

The study on nutritional practices in infants aged 7-12 months (the children aged 5 and 6 months were excluded, as it was uncertain whether they would still be exclusively breastfed) revealed that 54.1% of them were breastfed in the first 6 months of life, whereas the proportion of children exclusively breastfed in that period was 5.9%. Infant formula was introduced in the first month of life in 27.3% of infants. Table IV presents the proportion of children who received different food products in the initial phase of complementary feeding introduction. A significant number of parents (61.1%) started to expand the diet of their child before the fifth month of life. Water and teas for infants were the first nondairy products to be given to drink, followed by gluten-free baby cereals, fruit juices and fruit and vegetable puree. The diet of only 30.2% of the infants was expanded according to the recommendations, i.e. between 17 and 26 weeks of life (5-6 months of life).

**Table IV:** Introduction of infant formula and complementary foods into the diets of infants. Tabela IV. Wprowadzanie mleka modyfikowanego i żywności uzupełniającej do diety niemowląt.

No Lp.	Range of products *Asortyment produktów*	Percentage of infants (n=447) who had complementary foods introduced in consecutive months of life *Odsetek niemowląt (n=447), u których wprowadzono żywność uzupełniającą w kolejnych miesiącach życia*					
		1 month of life *1. m.ż*.	2 month of life *2. m.ż*.	3 month of life *3. m.ż*.	4 month of life *4. m.ż*.	5 month of life *5. m.ż*.	6 month of life *6. m.ż*.
1.	Infant formula *Mleko modyfikowane*	27.3	10.7	9.2	6.3	3.8	4.9
2.	Gluten-free infant cereals (rice, corn) *Kleiki, kaszki bezglutenowe (ryżowa, kukurydziana)*	0.9	1.3	5.1	24.8	21.0	13.4
3.	Gluten-containing infant cereals (semolina, wheat, multigrain) *Kleiki*, *kaszki* *zawierające gluten (manna, pszenna, wielozbożowa)*	0.4	1.1	2.9	11.4	17.7	16.3
4.	** Fruit juices *Soki owocowe*	0.2	0.7	2.9	21.0	14.3	14.5
5.	Water *Woda*	25.3	10.3	12.5	13.9	12.3	9.6
6.	Tea for children *Herbatka dla dzieci*	16.1	6.5	7.4	12.3	8.1	5.1
7.	Tea / *Herbata*	0.9	2.7	1.8	4.9	3.6	3.6
8.	** Crust/bread/roll *Skórka chleba/chleb/bułka* ** **	0.0	0.4	0.4	5.8	8.5	13.2
9.	** Fruit purees *Przeciery owocowe*	0.0	0.0	2.7	30.6	23.9	14.8
10.	** Purees/vegetable soups *Przeciery/zupki warzywne*	0.2	0.0	1.3	30.9	28.9	17.4
11.	Meat *Mięso*	0.2	0.0	0.2	7.8	20.8	21.3
12.	Fish *Ryby*	0.0	0.0	0.2	3.4	13.2	16.6
13.	Egg yolk *Żółtko*	0.0	0.0	0.2	3.1	4.7	15.9
14.	Whole egg *Całe jajo*	0.0	0.0	0.0	1.8	2.2	7.8
15.	Yoghurt/cottage cheese/cheese ** *Jogurt/twarożek/sery* **	0.2	0.0	0.2	5.4	8.1	12.3
16.	** Cow’s milk *Mleko krowie*	0.2	0.4	0.0	0.9	2.5	1.1
17.	Biscuits/sponge fingers *Herbatniki/biszkopty* **	0.0	0.0	0.9	6.7	11.2	15.7

Children in their second 6-month period of life received a varied diet in terms of food selection, its texture and energy value. Almost two-thirds (61.3%) of the children received meals cooked separately for them and 27.5% ate the same as the entire family every day or at least 2-4 times a week. Mothers used ready-to-serve foods intended for infants and young children (baby food).

Almost 90% of the children received such products every day or at least 2-4 times a week. Most frequently it was infant formula and baby cereals.

The data obtained show that the feeding practices in children in their first year of life do not meet the guidelines presented in the model of safe nutrition, particularly in matters of timing of complementary feeding introduction and food choice.

### Postinfancy

The body weight of 67.8% of the children aged 13-36 months was normal and 4.1% of children were underweight (tab. V). The high proportion of overweight children and those at risk of being overweight (28.1%) is noteworthy.

**Table V:** Nutritional status of the children aged 13-36 months defined by the weight-for-height ratio – nation-wide, Polish, representative sample. Tabela V. Stan odżywienia badanych dzieci w wieku 13-36 miesięcy określony poprzez znormalizowany wskaźnik masa ciała do długości/wysokości ciała – próba ogólnopolska, reprezentatywna

Nutritional status *Stan odżywienia*	Cut-off points Weight-for-height z-score (acc. to WHO) *Punkty odcięcia Weight-for-height z-score (wg WHO)*	Children aged 13-36 months (n=612) *Dzieci 13-36 miesięcy (n=612)*	
		N	%
** Possible ** risk ** of overweight *Możliwe ryzyko nadmiaru masy ciała*	>1 SD to 2 SD	113	18.4
Overweight *Nadwaga*	>2 SD to 3 SD	42	6.9
Obese Otyłość	>3 SD	17	2.8
Total *Łącznie*		172	28.1
Wasted *Niedobór masy ciała do długości /wysokości ciała*	<-2 SD to -3 SD	14	2.3
Severely wasted *Znaczny niedobór masy ciała do długości /wysokości ciała*	<-3 SD	11	1.8
Total *Łącznie*		25	4.1
** Normal nutritional status *Prawidłowy stan odżywienia* **	≥-2 SD to +1 SD	415	67.8

Approx. 10% of the children aged 13-36 months were still breastfed. According to the statement of the mothers, the average number of breastfeeding episodes was 6, including 2 at night. The average number of meals consumed by children during the day was at least 5. What is noteworthy is the significant proportion of children receiving different sorts of snacks between the main meals (tab. VI). The adequate arrangement of meals received by the children during the day ensures the child receives appropriate energy supply and prevents nutritional mistakes from occurring. Young children should receive 4-5 meals per day: 3 larger and 1-2 small ones but some of them may require a larger number of meals, yet smaller in volume.

**Table VI:** Feeding practices in children aged 13-36 months (n=612) – meal organisation. Tabela VI. Sposób żywienia dzieci w wieku poniemowlęcym (n=612) – organizacja posiłków.

Meals *Posiłki*	Percentage of children aged 13-36 months who consume such meals every day or at least 2-4 times a week *Odsetek dzieci w wieku 13-36 m.ż. spożywających różne posiłki codziennie lub przynajmniej 2-4 razy w tygodniu*
**Breastfeeding *Karmienie piersią***	10.0
**Recommended meals *Posiłki zalecane***	
breakfast *śniadanie*	99.3
second breakfast *drugie śniadanie*	93.5
dinner [soup/main course] *obiad [zupa/drugie danie]*	95.8/94.9
afternoon snack *podwieczorek*	94.0
supper *kolacja*	98.9
**Additional meals *Posiłki dodatkowe***	
bedtime meal *posiłek przed snem*	55.6
eating/drinking at night *jedzenie/picie w nocy*	42.0
snacking between meals *pojadanie*	85.1
**Feeding type *Forma żywienia***	
family meals *posiłki stołu rodzinnego*	87.4
meals prepared separately for the child *posiłki przygotowywane osobno dla dziecka*	26.3
meals based on baby foods intended for infants and young children *posiłki na bazie żywności gotowej dla niemowląt i małych dzieci*	14.5-43.0
meals not prepared at home *posiłki przygotowywane poza domem*	3.3

Table VII presents the comparison of an average daily food ration of the children aged 13-36 months with model food ration. We observed insufficient consumption of milk and fermented milk beverages, vegetables and fruits as well as fish, which results in an unbalanced nutrient profile of the toddlers’ diet.

**Table VII:** Average daily food ration of children aged 13-36 months (n=612) in relation to the model food ration. Tabela VII. Przeciętna całodzienna racja pokarmowa dzieci w wieku poniemowlęcym (n=612) w odniesieniu do modelowej racji pokarmowej.

	Group of products *Grupa produktów*	Unit *Jednostki*	Model food ration for children aged 13-36 months *Modelowa racja pokarmowa dla dzieci 13-36 miesięcy*	Consumption in group of children aged 13-36 months (n=612) *Spożycie w badanej grupie dzieci 13-36 miesięcy (n=612)*		Percentage of children with consumption below recommendations Odsetek dzieci ze spożyciem poniżej normy
				Median *Mediana*	Range 1-3 quartile *Zakres 1-3 kwartyl*	
1.	Cereal products and potatoes *Produkty zbożowe i ziemniaki*					
	** bread *pieczywo mieszane*	g	20	45.0	25.0-63.3	16.3
	flour, pasta *mąka, makarony*	g	25	19.3	11.4-32.8	62.6
	groats, rice, cereals *kasze, ryż, płatki śniadaniowe*	g	30	17.9	8.1-32.5	72.2
1A.	Potatoes *Ziemniaki*	g	100	70.8	41.8-116.3	66.7
2.	Vegetables and fruits *Warzywa i owoce*	g	450	277.6	195.3-384.2	82.7
	vegetables *warzywa*	g	200	100.8	62.1-158.0	87.9
	fruits *owoce*	g	250	170.3	99.1-251.9	74.3
3.	Milk and milk products *Mleko i produkty mleczne*					
	milk and fermented milk products *mleko i mleczne napoje fermentowane*	g	550	309.2	172.2-455.7	88.1
	including liquid milk *w tym mleko* *płynne*:	g	450	270.3	133.0-426.3	78.3
	cow’s milk *spożywcze (krowie)*	g	--	103.5	41.5-227.8	--
	infant formula *modyfikowane*	g	--	0.0	0.0-270.0	--
	fermented milk products *mleczne napoje fermentowane*	g	100	20.7	0.0-53.3	91.4
	** cottage cheese *sery twarogowe*	g	10-15	13.3	2.1-43.3	43.6
	cheese *sery podpuszczkowe*	g	2	0.0	0.0-5.7	59.6
4.	Meat, cured meat, fishes and eggs *Mięso, wędliny, ryby oraz jaja*					
	meat, poultry meat, cured meats *mięso, drób, wędliny*	g	20	68.8	43.8-100.5	7.5
	Fish *Ryby*	g	10	0.0	0.0-5.7	77.5
4A.	Eggs *Jaja*	g	25	22.7	6.8-41.3	53.9
5.	Fats *Tłuszcze*	g	16	17.2	10.8-25.6	44.6
	Butter and cream *Zwierzęce: masło i śmietana*	g	6	9.7	6.0-15.9	25.0
	Vegetable oils *Roślinne: oleje*	g	10	6.6	3.3-10.9	71.7
6.	Sugar and sweets *Cukier i słodycze*	g	20	25.3	13.3-40.1	39.9

The share of food products of special nutritional purpose, i.e. ready-to-serve food intended for infants and small children depended on the age of the children. Half of the children in the second year of life consumed a junior formula and baby cereals, one-third ate fruit purees and desserts and one-quarter of the group received ready-to-eat soups or dishes every day or at least 2-4 times a week. The diet of children in the third year of life was mainly the family diet with a significant share of wheat bread, pasta and breakfast cereals, as well as milk, fruit yoghurts and dairy desserts, poultry and cured meats, vegetables and fruits (tab VIII).

**Table VIII:** Feeding practices in children at the age 2 and 3 years. Tabela VIII. Sposób żywienia dzieci w 2. i 3. roku życia.

Eating frequency (every day or 2-4 twice a week) *Częstość spożycia (codziennie lub 2-4 razy w tygodniu)*	Percentage of children at the age of 2 and 3 years *Odsetki dzieci w 2. i 3. roku życia*		Children aged 13-36 months total (n=612) *Dzieci 13-36 miesięcy ogółem (n=612)*
	*2 nd year of life 2. rok życia (n=306)*	*3 rd year of life 3. rok życia (n=306)*	
Meals prepared separately for the child ** ** *Posiłki przygotowywane osobno dla dziecka*	37.6	14.7	26.3
** Family ** meals *Dieta stołu rodzinnego*	80.3	94.8	87.4
** Infant formula *Mleko modyfikowane*	56.7	29.0	43.0
Infant cereals *Kaszki/kleiki*	48.8	26.1	37.6
Baby food [soups/dishes] *Zupki/obiadki*	22.9	5.9	14.5
** Purees/fruit ** desserts *Przeciery/deserki owocowe*	31.7	11.4	21.7
** Wheat bread *Pieczywo pszenne*	84.8	86.9	85.8
Pasta *Makarony*	51.4	54.3	52.8
** Breakfast cereals *Płatki śniadaniowe*	33.9	49.4	41.5
Cow’s milk *Mleko płynne spożywcze*	41.9	62.1	52.0
Fruit yoghurts, dairy desserts *Jogurty owocowe, desery mleczne*	57.7	67.6	62.6
Poultry *Drób*	92.9	91.8	85.5
Cured meats *Wędliny*	78.0	82.4	80.1
Vegetables *Warzywa*	83.7	84.3	84.0
** Fruits Owoce	92.9	91.8	92.3

The analysis of the nutrient profile in toddlers’ diets indicated the differentiated energy and protein intake was significantly higher than population norms (EAR/ AI) (tab. IX). In 74.8% of children the share of energy originating from sucrose was greater than recommended (% of energy from sucrose <10). The diet of almost every child contained a shortage of long chain polyunsaturated fatty acids (LCPUFA), vitamin D and potassium (99.0%, 94.4% and 87.4% respectively). Insu? cient intake of fats, vitamin E, calcium and fibre was observed in every second child, whereas a shortage of energy and iodine occurred in almost every third child. The data obtained confirm the significant diversity of the nutritional value of diets, which in turn indicates the need for their monitoring. Insufficient intake of long chain polyunsaturated fatty acids (LC PUFA) and vitamin D by children justifies the need for the supplementation of these nutrients. Other insu? ciently supplied nutrients should be delivered with adequately selected food from different groups.

**Table IX:** Nutrient profile in the diet of children aged 13-36 months in relation to nutritional recommendations (n=612). Tabela IX. Profil składników odżywczych w dietach badanych dzieci w wieku poniemowlęcym w odniesieniu do norm żywienia (13-36 miesięcy życia; n=612).

Nutrients *Składniki pokarmowe*	Unit *Jednostka*	Children aged 13-36 months (n=612) *Dzieci 13-36 miesięcy (n=612)*		Recommendation EAR/AI Norma EAR/AI*	Percentage of children consuming less than recommedations *Odsetek dzieci* *ze spożyciem poniżej normy*
		Median *Mediana*	1-3 quartile *1-3 kwartyl*		
Energy *Energia*	kJ	4638.0	3826.1-5693.4	--	
Energy *Energia*	kcal	1105.2	913.6-1355.9	1000	36.6
** Total protein *Białko ogółem*	g	40.7	32.1-50.6	12	0.3
Fat *Tłuszcz*	g	36.3	28.6-46.7	39	58.7
LCPUFA *LCPUFA*	mg	37.1	18.5-76.5	250*	99.0
Total carbohydrates *Węglowodany ogółem*	g	161.2	131.2-195.1	--	--
Digestible carbohydrates *Węglowodany przyswajalne*	g	152.4	123.7-183.7	100	11.1
Sucrose *Sacharoza*	g	38.2	25.6-53.7	--	--
Lactose *Laktoza*	g	15.7	8.6-26.6	--	--
** Starch Skrobia	g	56.4	42.0-75.1	--	--
Dietary fibre *Błonnik pokarmowy*	g	9.6	7.4-12.4	10*	56.4
Percent of energy ** from protein *Procent energii z białka*	%	14.6	12.9-16.7	--	--
Percent of energy ** from fat *Procent energii* *z tłuszczu*	%	29.6	26.1-33.2	--	--
Percent of energy from carbohydrates *Procent energii z węglowodanów*	%	55.5	51.2-59.9	--	--
Percent of energy ** from sucrose *Procent energii z sacharozy*	%	13.9	10.0-18.0	<10%	25.2
Minerals *Składniki mineralne*					
Sodium *Sód*	mg	1541.8	1122.9-1944.9	750*	9.3
Ptassium *Potas*	mg	1711.3	1361.7-2110.2	2400*	87.4
Calcium *Wapń*	mg	546.7	414.2-707.8	500	42.3
Phosphorus *Fosfor*	mg	685.8	556.0-855.4	380	5.4
Magesium *Magnez*	mg	150.2	119.0-187.6	65	2.0
Iron / *Żelazo*	mg	6.98	5.35-8.85	3	2.0
Zinc *Cynk*	mg	5.68	4.44-6.72	2.5	2.0
Cooper *Miedź*	mg	0.60	0.46-0.74	0.25	1.1
Manganese *Mangan*	mg	1.66	1.16-2.20	--	--
Iodine *Jod*	μg	88.9	65.1-112.5	65	27.5
Vitamins *Witaminy*					
Vitamin A (retinol equivalent) *Witamina* *A (ekwiw. retinolu)*	μg	829.8	585.4-1155.7	280	3.4
Vitamin E (alpha-tocopherol equivalent) *Witamina E (ekwiw. alfa-tokoferolu)*	mg	5.6	4.0-7.5	6*	59.2
Thiamine *Tiamina*	mg	0.73	0.57-0.98	0.4	7.0
Riboflavin *Ryboflawina*	mg	1.15	0.90-1.44	0.4	1.8
Niacin *Niacyna*	mg	8.60	6.78-11.26	5	10.1
Vitamin B_6_ *Witamina B*_6_**	mg	1.07	0.86-1.35	0.4	1.1
Vitamin B_12_ *Witamina B*_12_**	μg	2.00	1.50-2.77	0.7	3.1
Vitamin D *Witamina D*	μg	3.49	1.43-6.36	10	94.4
** Vitamin C *Witamina C*	mg	83.1	54.0-118.1	30	7.5
Folate (diet equivalent) *Foliany* *(ekwiw*. *diety)*	μg	161.1	127.5-201.1	120	20.8
** Folic acid *Kwas foliowy*	μg	7.7	0.0-39.3	--	

## Discussion

According to the guidelines of the World Health Organisation (WHO) and European nutrition societies, exclusive breastfeeding is the optimal method of feeding infants for 6 months followed by the appropriate complementary feeding introduction in the second half of their first year of life [11, 12, 20, 21]. Breastfeeding ensures that all the nutritional requirements of the child are met and optimally stimulates his/her development. The qualitative and quantitative composition of mother’s milk is ideally adjusted to the requirements of the infant. Apart from vitamin D and K, this applies to all nutritional elements, including proteins. It was shown that breastfeeding of babies from their birth till the age of 2 years is associated with a lower intake of protein and reduced risk of being overweight later in life, when compared to formula-fed children [4]. The model of safe infant nutrition emphasises the fact that exclusive breastfeeding is an optimal feeding method in the first six months of life, whereas nutrition during the second half of the first year of life should be based not only on breastfeeding but also on incorporating complementary foods according to recommendations [10, 20].

In the first 6 months of life 54.1% of infants from the studied group were breastfed, including 5.9% exclusively. The data obtained are similar to those gathered during epidemiological studies carried out by other authors [14]. The studies performed in 2014 evaluated the nutrition of infants (n=1679) and revealed that 38% of them were breastfed in their sixth month of life, 4% of whom exclusively [22]. These data differ from the results of previous studies performed in Poland and other European countries, which indicated a greater percentage of exclusively breastfed children (13-14%) [14, 23]. The question remains, however, why despite the widespread promotion of natural feeding presented during antenatal classes and with the use of different educational forms − the rate of breastfeeding women remains on a similar level. This issue requires separate studies.

Since the sixth month of life natural feeding or that carried out with the use of infant formula no longer provides an adequate supply of energy, protein, iron, zinc and some vitamins. Therefore, the introduction of complementary foods is necessary. According to current recommendations, it is considered that complementary foods should be introduced no sooner than in the 17th week of life, but no later than in the 26th week of life [21].

According to the ESPGHAN guidelines, exclusive or full breastfeeding should be promoted at least until the end of the fourth month of life (17 weeks), whereas exclusive or predominant breastfeeding for approx. six months of life (26 weeks) [21]. It is recommended that complementary foods (fluids and solid foods other than mother’s milk and infant formula) should not be introduced before the age of 4 months and delayed beyond 6 months. The diet should be supplemented with products of diverse texture and taste, including green vegetables with a bitter taste. Breastfeeding should be continued alongside with the introduction of complementary foods. Small amounts of cow’s milk should be used to prepare complementary foods, yet it should not be used as the main milk product until the age of 12 months. Potentially allergenic food may be introduced when the complementary feeding is commenced (but not before the fifth month of life), yet such practice requires supervision by a specialist. Gluten-containing products may be introduced between the 4 and 12th months of age. Nevertheless, it should be noted that their amount should be limited during the first few weeks. All infants should receive iron-fortified foods (e.g. cereal products) and food being a natural source of iron (meat). Adding salt and sugar to the products and meals intended for children should be avoided, as well as drinking sweet beverages. The consumption of fruit juices should be limited. Vegan diets require the supervision of a paediatrician and/or dietician and appropriate supplements. The parents should be aware of the health consequences of such a diet. Parents’ alertness to nutritional requirements of their child, signalled as a feeling of hunger or satiety, is equally important. It has been proven that such a practice is important from the nutritional, developmental and health point of view, because it is associated with a significant reduction of the risk of contracting infectious, especially gastrointestinal and respiratory, allergic and autoimmune diseases. Moreover, it has no negative impact on the rate of growth, body composition and does not increase the risk of becoming overweight or obese [21].

In the studied group 61.1% of the children received complementary feeding earlier than it is recommended (before the 5th month of life) and only 30.2% of the infants received their first non-dairy foods (baby cereals, vegetable and/or fruit purees, juices, infant teas) between the 17th and 26th week of life. These results unequivocally indicate that almost two-thirds of the mothers of the studied children did not know the optimal age for the introduction of complementary foods. Therefore, it is necessary to monitor the feeding practices of infants and to provide nutritional counselling for the mothers of young children alongside routine inpatient paediatric care.

The energy and nutrient intake in children’s diet should meet nutritional recommendations [19]. The protein requirement of children was estimated at approx. 1 g/kg body mass, yet it should not exceed 15% of the total recommended energy intake (1000 kcal/day). Fats should deliver 20-35% of total energy, so that they cover the energy expenditure of the child, including the part required for growth. The intake of fat of adequate quality is very important as well, including the sources of fatty acids, especially long chain polyunsaturated fatty acids (LCPUFA). Carbohydrates should account for 55-60% of energy. The amount of added sugars should be limited (less than 10% of total energy). Products delivering complex carbohydrates should be preferred in the diet of children. Recommended dietary allowance (RDA) for calcium in a 1-3 year old child is 700 mg/day, while the population estimated average requirement (EAR) is 500 mg/day. According to medical standards, a child requires approx. 15 Zg (600-1000 IU) vitamin D_3_/day [5, 19, 24].

When it comes to the group of children over 1 year old, it is worth noticing that 10% of this group were still breastfed and 43.3% were receiving junior formula, whereas the proportion of children drinking such formula decreased significantly after the 18th month of life. The share of junior formula in the nutrition of children aged 1 year and older significantly impacts the nutritional value of their diet [25]. The adequate supply of energy and nutrients, especially proteins, DHA, vitamin D, iodine and iron still significantly in$uences the processes associated with the metabolic and nutritional programming in a child, and reduces the long-term risk of diet-related diseases, including obesity [25]. The nutrient profile in the analysed diets of children did not meet the recommendations. The results showed insu? cient intake of many nutrients important for the normal development of the child, i.e. LCPUFA, vitamin D and potassium. Furthermore, the increased risk for energy, fat, fibre, vitamin E and calcium deficiency was observed in the diet of almost every second or third child. Such results prove the poorly diversiThed selection of products used in nutrition of the youngest children. The research of other authors, who analysed the quantitative and qualitative composition of children’s diets, revealed a similar trend [8, 9, 26, 27, 28]. These data confirm that it is necessary to popularise the model food ration for children aged 1-3 years among parents. It seems that the results obtained should provide the basis for reformulation of the quantitative and qualitative composition of dietary products/food intended for infants and young children.

The population study conducted on the group of children aged 5 to 36 months indicates the problem of excessive intake of sucrose and salt in children’s diet. The WHO mission for 2015-2020 emphasises the necessity to reduce the amount of such nutrients in children’s nutrition [29].

Therefore, it is necessary to develop such forms of educational impact for the parents of young children, which will eventually change their attitude towards healthy nutrition.

## Conclusions

1. It is necessary to modify infants’ and young children’s nutrition through a better selection of products.
2. Nutritional practice should always be monitored and modified according to the model of safe nutrition as a part of medical nutritional counselling.
3. More educational efforts are required to increase the awareness of the relation between diet and the nutritional status of young children among healthcare professionals.
